# The Effect of Titanium Tetra-Butoxide Catalyst on the Olefin Polymerization

**DOI:** 10.3390/polym13132109

**Published:** 2021-06-26

**Authors:** Mohammed S. Alsuhybani, Eid M. Alosime

**Affiliations:** King Abdulaziz City for Science and Technology (KACST), P.O. Box 6086, Riyadh 11442, Saudi Arabia; sohybani@kacst.edu.sa

**Keywords:** polyethylene, olefin polymerization, Ziegler–Natta, polyvinyl chloride

## Abstract

The purpose of this study was to assess the ability of titanium Ti(IV) alkyloxy compounds supported by organic polymer polyvinyl chloride (PVC) to polymerize ethylene by feeding triethylaluminium (TEA) as a cocatalyst. Additionally, the impacts of the molar ratio of [Al]/[Ti] on the catalytic activities in ethylene’s polymerization and of the comonomer through utilization of diverse quantities of comonomers on a similar or identical activity were studied. The optimal molar ratio of [Al]/[Ti] was 773:1, and the prepared catalyst had an initial activity of up to 2.3 kg PE/mol Ti. h. when the copolymer was incorporated with 64 mmol of 1-octene. The average molecular weight (*M*_w_) of the copolymer produced with the catalysts was between 97 kg/mol and 326 kg/mol. A significant decrease in the *M*_w_ was observed, and PDI broadened with increasing concentration of 1-hexene because of the comonomer’s stronger chain transfer capacity. The quick deactivation of titanium butoxide Ti(OBu)_4_ on the polymers was found to be associated with increasing oxidation when supported by the catalyst. The presence of Ti(III) after reduction with the aluminum alkyls cleaves the carbon-chlorine bonds of the polymer, producing an inactive polymeric Ti(IV) complex. The results show that synergistic effects play an important role in enhancing the observed rate of reaction, as illustrated by evidence from scanning electron microscopy (SEM). The diffusion of cocatalysts within catalytic precursor particles may also explain the progression of cobweb structures in the polymer particles.

## 1. Introduction

Ziegler-Natta (ZN) catalysts are the most commonly used catalysts in the olefin polymerization industry [1]. They are mainly composed of transition metal compounds, such as titanium, chromium, and vanadium precursors [2], and they are considered the best option for olefin polymerization industries because of their high productivity and good morphology control [3]. They undergo activation through the use of either an activator or a cocatalyst that activates inactive sites [4]. The most commonly used cocatalysts are alkylaluminium based, such as triethylaluminium (TEA) and tri-octyl aluminum [5,6]. 

MgCl_2_ in combination with either TiCl_4_ or TiCl_3_ enhances the effectiveness of ZN catalysts and TEA cocatalysts [7,8]. In the synthesis of a novel chromium SiO_2_/MgO-based ZN catalyst using water-soluble magnesium sources, the Cr-Ti catalysts have been reported to increase the polymerization activity and can generate polyethylene with a favorable hydrogen response [9]. Kinetic investigations of ethylene polymerization have demonstrated that two types of active sites, TiCl_4_ and Cp_2_ZrCl_2_, are formed when TiCl_4_ and zirconocene (Cp_2_ZrCl_2_) catalysts are anchored with a MgCl_2_(THF)_2_ support and then activated using TEA and methylaluminoxane [10]. Furthermore, silicon dioxide (SiO_2_) has also been utilized as a support [11]. 

A MgCl_2_/SiO_2_ bisupport utilizes magnesium acetate as a source of Mg. When MgCl_2_/SiO_2_ reacts with TiCl_4_ and VOCl_3_ under different reaction sequences, ZN hybrid titanium/vanadium catalysts are formed [12]. The effectiveness of the Ti/V hybrid catalysts lies between that of the MgTi/Si and MgV/Si catalysts. However, the Ti/V hybrid catalysts, which result from a coreaction with TiCl_4_ and VOCl_3_, show increased activity compared with the Ti/V hybrid catalysts prepared using a two-step mechanism [13].

The polymerization rate is influenced by factors such as the concentration of the active center, the propagation rate constant, and the monomer concentration [14]. The concentration of the monomer changes gradually as one moves from the surface toward the core of the polymer/catalyst particles [15,16]. The concentration of the monomer is assumed to remain even across the entire polymer medium, whereas the propagation rate constant highly depends on the stereospecificity of the active centers [17]. The increased concentration of the active center promotes the activity of the ZN catalysts [18]. In addition, an appropriate amount of diethylaluminium chloride is added to the ZN catalyst system. Here, alongside TEA as the cocatalyst, the catalytic activity increases [19].

The functional groups within polymer structures play a crucial role in promoting the formation of either chemical bonds or interactions between the polymers and catalysts [20]. However, polymers such as atactic poly(propylene), natural rubber, and polyvinyl chloride are characterized by low-surface free energies and a lack of functional grouping. Therefore, they cannot interact with ZN catalysts unless functional groups are introduced [21]. Catalytic systems containing chlorine either as part of the support or as a cocatalyst exhibit enhanced activity; therefore, to allow for the study of their activities, PP, NR, and PVC are chlorinated before being subjected to the heterogenization of the ZN catalyst [22]. 

Recently, it has been discovered that the use of Ti(IV) alkoxide complexes—with 1,2- and 1,4-diolate ligands activated by a binary activator {Bu_2_Mg + Et_2_AlCl} and as a catalyst during the polymerization process of ethylene—results in the formation of an ultrahigh molecular weight polyethylene (UHMWPE) [23]. Ti(IV) complexes with diolate ligands are also very efficient in the copolymerization of ethylene and α-olefins. However, the effectiveness of the various diolate complexes differs based on the size of the chelate ring and the amount of aluminum chloride used [23].

In the current paper, we present our experiments, which are aimed at further understanding ZN catalysts and the processes for olefin polymerization. The titanium tetra-butoxide Ti(OBu)_4_ compound was used as a compound-containing oxygen to determine the impact on the TiCl_4_ catalyst, which was supported using PVC polymeric material. The latter was treated by a Grignard compound, and TEA (Al(C_2_H_5_)_3_) was used as a cocatalyst. The utilization of a PVC-based polymeric support in preparing the catalyst provided important benefits compared with contemporary methods that use polymerization catalysts supported by magnesium chloride (MgCl_2_) and SiO_2_. Moreover, the PVC-based polymeric supports (or particles) for preparing the catalysts have shortened the dehydration duration and steps, allowing for lower temperatures compared with the polymerization catalysts supported by MgCl_2_ and SiO_2_. Therefore, PVC-based polymeric supports (or particles) are more suitable for synthesizing catalysts because they simplify the synthesis process, leading to a significant reduction in the cost of preparing catalysts. Additionally, the cost of PVC-based support tends to be considerably lower compared with polymerization catalysts that are supported by MgCl_2_ and SiO_2_. Similarly, the catalyst substantially utilizes the lowest levels of catalyst precursors in preparing the catalyst compared with the polymerization catalysts supported by MgCl_2_ and SiO_2_. The catalytic activities of Ti(OBu)_4_ catalysts, for both ethylene homopolymerization and its copolymerization, ethylene, and 1-octene and ethylene and 1-hexene, were assessed.

## 2. Experiment

### 2.1. Materials

Ethylene gas was supplied from Abdullah Hashem Co., Saudi Arabia, with a purity of 99.95%. Here, *n*-hexane served as a polymerization medium in the heterogenous phase and was purchased from BDH^®^ with a purity of 99%. In addition, 1-octane was purchased from Ried Dehean, and 1-hexene was obtained from Advanced Engineering, UK, and dried over 5 Å molecular sieves before use. Butylmagnesium chloride (BuMgCl) was purchased from Aldrich Chemical (2 M in THF). Titanium(IV) butoxide (Ti(OBu)_4_) with 100% purity was purchased from Akzo Chemie America. PVC spheres with an average particle size of 50 μm were used (supplied by SABIC, Riyadh, Saudi Arabia). All support and catalyst synthesis and characterizations were performed under inert gas.

### 2.2. Synthesis of PVC/BuMgCl/Ti(OBu)_4_∙TiCl_4_ Catalyst 

The PVC/BuMgCl/Ti(OBu)_4_∙TiCl_4_ catalyst was synthesized using a three-neck round-bottom flask equipped with a condenser and stirrer. Here, 5.0 g of PVC was added into the flask, and the flask containing the PVC was heated to 90 °C using an oil bath for 30 min. Then the flask was evacuated at 40 mbar pressure for 30 min. The flask and its contents were purged with dried nitrogen three times to ensure that the flask did not contain any oxygen, and the PVC was slurried using 30 mL of n-hexane. Afterwards, 1.5 mL of 2 M BuMgCl was added to the slurry, and the resultant mixture was stirred for 5 min at 60 °C. After this, 0.2 mL of 1 M Ti(OBu)_4_ was added to the previous slurry, and the resulting mixture was stirred for 60 min at 60 °C. Once it was swept using nitrogen for a period of 60 min, the resultant precursor was then vacuum dried at 110 °C for 30 min. The obtained powder was then mixed with 2 mL of TiCl_4_ (1 M) followed by stirring for 1 h. The mixture was brown before the participate distilled out; the hexane was then removed by cannula. The mixture was washed with 30 mL n-hexane three times and then dried and purged with nitrogen. A solid brown spherical catalyst particle was obtained. The contents of Ti and Mg were determined as 0.000379 and 0.00507 mmol respectively using inductively coupled plasma spectroscopy. 

### 2.3. Ethylene Homopolymerization

Ethylene polymerization was tested at five different molar ratios of cocatalyst TEA to Ti [Al]/[Ti]: 309, 618, 773, 927, and 1236 mmol, to which 0.02 g of the catalyst was added before pressurization with ethylene.

Ethylene homopolymerizations were carried out in a 2 L glass jacketed reactor. The reactor was purged with a high-vacuum pump with nitrogen at 130 °C for 1 h to ensure that all moisture and oxygen were removed. Afterwards, 400 mL of n-hexane was added into the reactor. The reactor was stirred and heated to a reaction temperature of 88 °C. Ethylene was then introduced, followed by the TEA cocatalyst, which was left in for 5 min. The PVC/BuMgCl/Ti(OBu)_4_∙TiCl_4_ catalyst was then fed into the reactor, followed by ethylene gas to maintain a specific pressure of 7 bar for 1 h of the polymerization reaction. The reactor was maintained and controlled at the desired reaction temperature throughout the polymerization process. Upon completion, the ethylene flow was stopped, and the reactor pressure was gradually lowered through venting. Ethylene consumption was automatically recorded using a flowmeter. The reactor was opened, and the polymer product was collected, filtered, and washed with petroleum ether, methanol, and chloric acid before being dried overnight in a vacuum oven at 70 °C.

### 2.4. Ethylene/1-Octene and Ethylene/1-Hexene Copolymerization

Copolymerizations of ethylene/1-octene (32, 48, 64, 96, and 127 mmol of 1-octene) and ethylene/1-hexene (40, 60, 68, 80, and 160 mmol of 1-hexene) were carried out in n-hexane in a 2 L glass jacketed reactor equipped with a mechanical stirrer. The reactor was purged with a high-vacuum pump with nitrogen at 130 °C for 1 h to ensure that all moisture and oxygen were removed. The reactor was charged with 400 mL of 1-hexene. Next, the ethylene gas feed was started, followed by equilibration at the desired polymerization temperature. After 15 min, a solution of TEA cocatalysts in n-hexane was added and stirred for 5 min, and an n-hexane solution (1 mL) of the titanium complex PVC/BuMgCl/Ti(OBu)_4_∙TiCl_4_ was then added to the reactor with vigorous stirring to initiate polymerization. During the polymerization process, the mixture of ethylene copolymer was fed to the reactor continuously to maintain atmospheric pressure. Upon completion of the polymerization process, the ethylene flow was stopped, and the reactor pressure was slowly released through venting. Ethylene consumption was automatically recorded using a flowmeter. The reactor was opened, and the polymer product was collected, filtered, and washed with petroleum ether, methanol, and chloric acid before being dried overnight in a vacuum oven at 70 °C.

### 2.5. Characterization

The polymer features were investigated through differential scanning calorimetry (DSC), thermogravimetric analysis (TGA), gel permeation chromatography (GPC), and scanning electron microscopy (SEM). DSC measurements of the polymer samples were carried out using a differential scanning calorimeter (DSC-8500, PerkinElmer, Shelton, CT, USA). The sample weight was approximately 10 mg, and the employed heating rate was 10 °C/min. The sample was heated to 200 °C and allowed to rest for 2 min to remove the thermal history; it was then cooled down to 25 °C at 10 °C/min and finally heated to 200 °C at 10 °C/min to record the second heating curve and melting temperature (*T*_m_) because the first heating cycle is affected by the mechanical and thermal history of sample preparation. The enthalpy of fusion (∆H_f_) of each sample was also calculated using the DSC curve.

The thermal behavior of the mixing catalyst/cocatalyst molar ratio of the polymerization was evaluated using a thermogravimetric analyzer (TGA 1, Perkin Elmer, Shelton, CT, USA). Each sample was heated from room temperature to 700 °C at a rate of 10 °C/min. Thermal decomposition temperatures and weight loss were determined from the weight–temperature curve. The TGA test was performed under a nitrogen atmosphere.

The average molecular weight (*M*_w_) and polydispersity index (*M*_w_/*M*_n_) were measured using a gel permeation chromatograph (GPC 2000, Waters Corp., Milford, MA, USA) with standard polystyrene as a reference and 1,2,4-trichlorobenzene (TCB) as the solvent. Polymers were dissolved at 150 °C in TCB in a concentration of 1.0 mg/mL before injection into the chromatograph.

The morphological characteristics of the catalyst particles incorporated during polymerization and copolymerization were studied by SEM (JSM-IT300, Jeol, Japan).

In this experiment, high-temperature ^13^C NMR (Bruker Avance 400, Bruker Biospin Karlsruhe, Germany) was used to determine the incorporation of comonomers into respective polymers. About 100 mg of the sample was then equipped into an NMR tube (5 mm) with the organic solvent 1,4-dichlorobenzene-d_4_ with a sample concentration of approximately 15 mg/mL. This was then scanned using Varian Inova-400 MHz at 110 °C at 100.62 MHz having a delay of 3 s for 10 h. The internal reference for the polymer chain was considered to be the carbon backbone at 30 ppm. Subsequently, 1-octene and 1-hexene content were introduced and their contents calculated taking into consideration the normalized integration area within the ^13^C NMR spectrum as outlined in the study by Seger and Maciel [24].

## 3. Results and Discussion

### 3.1. Ethylene Homopolymerization and Characterization of Homopolymers

**Figure 1:**
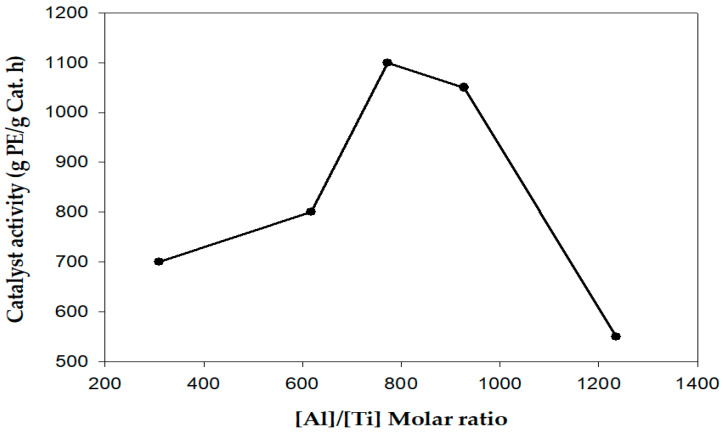
Variation of catalyst activity as a function of [Al]/[Ti] molar ratio in ethylene polymerization.

The use of the catalyst system PVC/BuMgCl/Ti(OBu)_4_∙TiCl_4_ in the polymerization of a single type of monomer to form a homopolymer was examined at various stimulus ratios to determine the best [Al]/[Ti] ratio resulting in high polymer productiveness. The catalyst molar ratio’s power to affect the activity of the catalyst and monomer inclusion as a function of the [Al]/[Ti] molar ratio in the polymerization process is summarized in Table 1. With only the [Al]/[Ti] molar ratio varying during the procedure, the catalyst amount was maintained at a constant 0.02 g. The optimum cocatalyst amount was influenced by the low [Al]/[Ti] ratio value. Considering the optimal conditions observed under the previous conditions (Figure 1), the molar ratio [Al]/[Ti] corresponds to 773, which represents a very high [Al]/[Ti] ratio. In addition, ethylene polymerization over PVC/BuMgCl/Ti(OBu)_4_∙TiCl_4_ is more economical than traditional heterogenous catalysts: as the activities increase, less catalyst will be required to produce the same amount of polymer in the polymerization reactor. As a consequence, there will be less residual catalyst in the product, thereby reducing the cost [25]. The catalytic activity decreased as the [Al]/[Ti] ratio increased from 927 to 1236, presumably because the prereduction influence of the alkyl aluminum reagent decreased the valence of the active site to an appropriate level and a somewhat higher dosage of cocatalyst brought about the deactivation from overreduction [13]. However, the necessity of having optimized processing conditions has decreased, which allows for a significant increase in the versatility of the process, making the commercialization of new polyolefin materials possible.

**Scheme 1:**
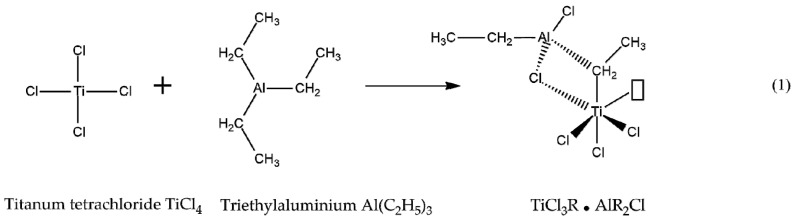
Chemical reaction of cocatalyst in Ziegler-Natta catalyzed olefin polymerization.

**Scheme 2:**
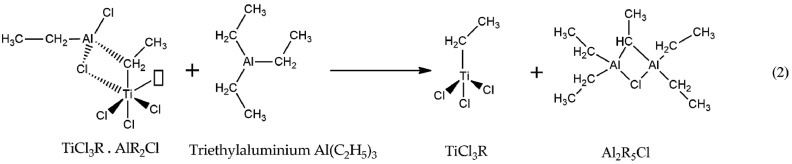
Ethylene polymerization reaction with heterogeneous Ti-based Ziegler-Natta catalyst, where R is Et.

In general, the TEA is known to alkylate the titanium species prior to the respective incoming alkene molecule coordination to neighboring vacant sites in the titanium. Chain elongation is thus produced by alkyl migration on the organic ligand thus leaving another vacant site for insertion of another molecule. The outlined insertion process is repetitive leading to the creation of a polymer chain. Eventually, the polymer chain is then hewed from the respective catalyst through the process of beta-hydrogen elimination [8]. An increase in the [Al]/[Ti] ratio for a small amount of catalyst explains the increase in catalyst activity. This reduces the number of activated sites, hence inducing the polymerization centers and leading to a decrease in polymerization activities, as stated in the kinetic model proposed by Hongrui et al. [26]. The importance of the presence of a cocatalyst to stimulate the active centers is its ability to polymerize, as in the following reactions (Scheme 1 and Scheme 2):

A fraction of the catalyst material will not be fully prepolymerized if its residence time in the prepolymerization reactor is too short. Indeed, too short a residence time can result in catalyst deactivation or the formation of undesired fines in the principal reactor. The prepolymerization in the dilute slurry phase of the batch operation introduces extra costs because of its discontinuity and requires separation of the inert hydrocarbon and the prepolymer material. Lack of catalyst activity with increasing concentrations of aluminum results from the increase in the cocatalyst. The presence of TEA leads to adsorption of the effective centers and competition between monomer molecules for adsorption onto these centers. As a result, the decrease in active centers does not favor polymerization. Furthermore, large quantities of cocatalyst reduce the oxidation state of the titanium atom in the active center. Consequently, there will be a weakening of activities in the active center, producing an ineffective center.

**Figure 2:**
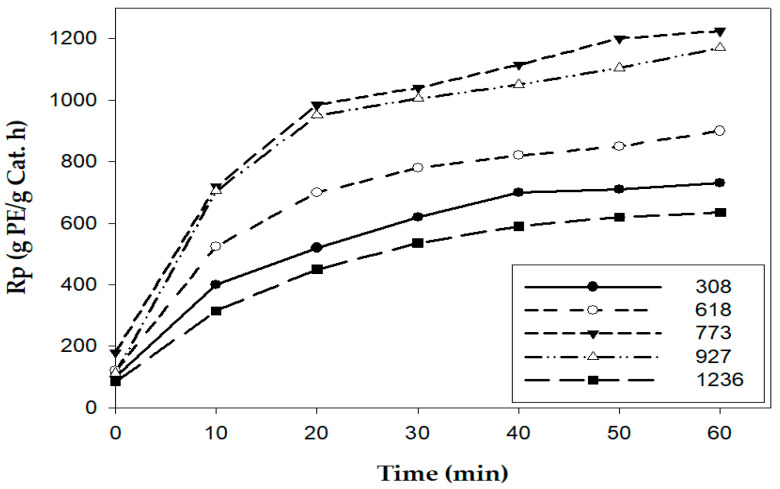
Corresponding rate profiles of the ethylene homopolymerization carried out using various [Al]/[Ti] molar ratios.

A systematic increase in the average velocity profiles for polymerization over time is the result of the slower process of alkylation. The rate profiles expressed as grams of polymers produced per hour and per gram of catalyst are presented in Figure 2. An [Al]/[Ti] ratio of 618 or lower was used for catalysis preparation, indicating a constant rise in activity over time. However, the rate profiles from the [Al]/[Ti] ratios of 773 and 927 show a rapid increase in the polymerization rate from the beginning to the end, reaching a peak and maintaining the status to the end. The opposite was seen for the [Al]/[Ti] ratio of 1236, in which the kinetic curve indicates that the maximum point was acquired within a short time. The change is proof that formation and active site deactivation are two independent processes [7]. The best productivity in the operation of the catalyst was achieved with a molar ratio of 773. Nevertheless, at a high yield (>2 g∙g^−1^), the catalyst portions, despite being small, likely drift toward the surface of the polymer particles [27]. Similar results were reported by Zheng et al. [15], who investigated the effect of [Al]/[Ti] catalytic activity and found that catalytic activity was high, as shown by the yield activity of Ti species. They considered TEA the strongest agent for alkylating titanium chloride because of the balance of the two active sites changed as the relative proportion of metal species was altered.

**Figure 3:**
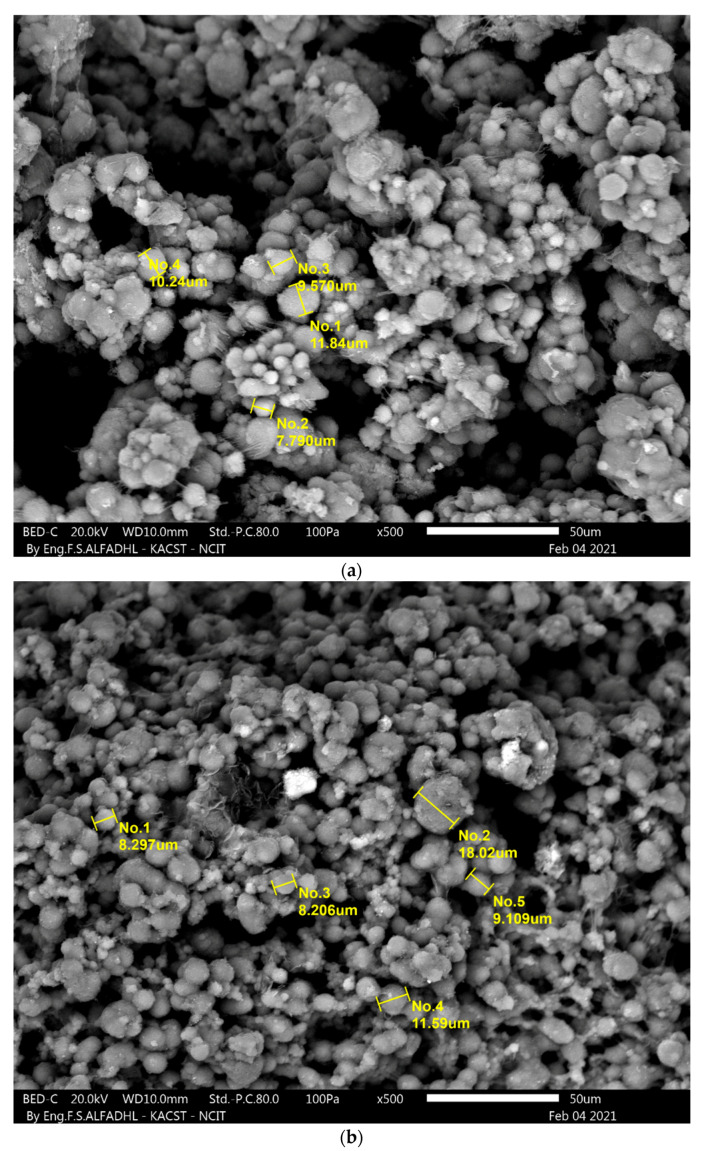

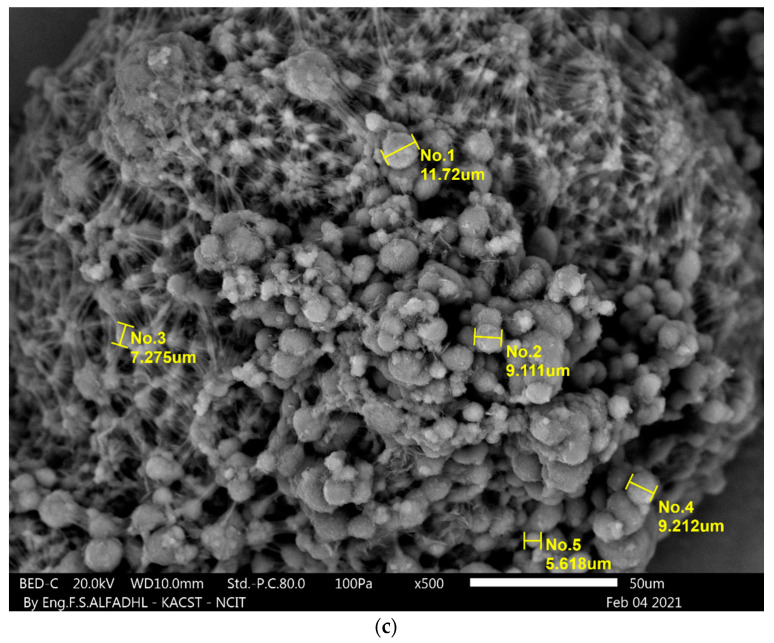
SEM result of polymer and catalyst particle in various [Al]/[Ti] molar ratios: (**a**) 309, (**b**) 773, and (**c**) 1236.

Figure 3 shows the SEM results for ethylene polymerization using various molar ratios of [Al]/[Ti]. (Details regarding the presence of all constituent atoms of the catalysts according to the analysis of EDS images are given in the Supplementary Materials, Figures S1–S5). It is evident that the catalyst particles remain in a good spherical shape and that the nodal structure blossoms. The increase in cocatalyst at a [Al]/[Ti] ratio of 1236 showed that the particles of the polymer differed greatly in size and were less porous and “fused.” The sample shows many fibrils. In addition, the current study found that the time necessary to attain maximum polymerization activity determined the structure of the polymer particles, in line with the observations of Nooijen [28]. The time required to reach the maximum polymerization activity is contingent on the rate of reaction between the catalyst precursor and the cocatalyst, which consequently depends on the type of cocatalyst. For instance, suppose it takes a long time to attain the maximum polymerization activity; the polymer particles will tend to show open cobweb structures [29]. The gradual increases in catalytic activity are the reason for the development of cobweb structures. This is because the increase in activity can cause the polymer to crack, leading to the formation of open structures with fibrils [28,29], as indicated in Figure 3c. 

**Figure 4:**
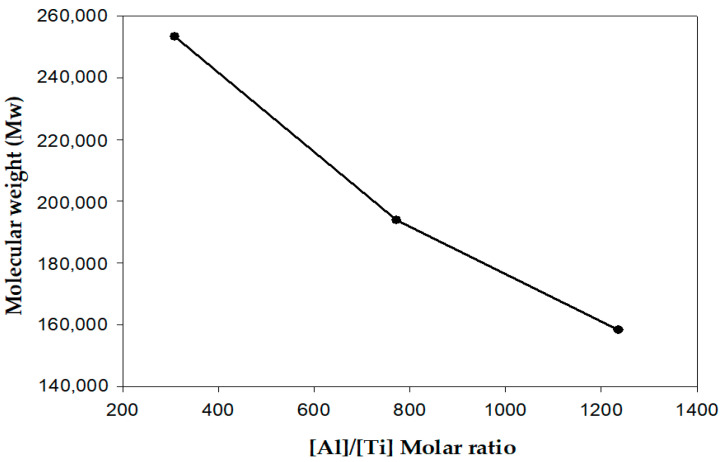
The relationship between the [Al]/[Ti] molar ratio and the molecular weight of polyethylene.

The data acquired from the GPC offer an estimate of the polymer *M*_w_, indicating that polymerization was affected by the [Al]/[Ti] ratio (Figure 4). An increase in the dosage of the cocatalysts is shown, which would contribute to the decrease in *M*_w_. The strong chain effect of alkyl metal salts can lower the *M*_w_ through the transfer of the nascent polymer chain of the active sites [30]. 

**Figure 5:**
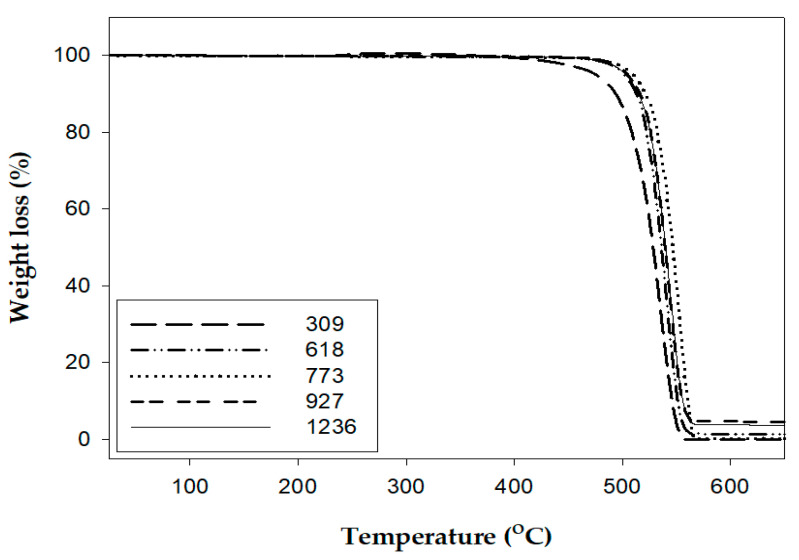
TGA thermograms of the ethylene homopolymerization carried out using various [Al]/[Ti] molar ratios.

**Figure 6:**
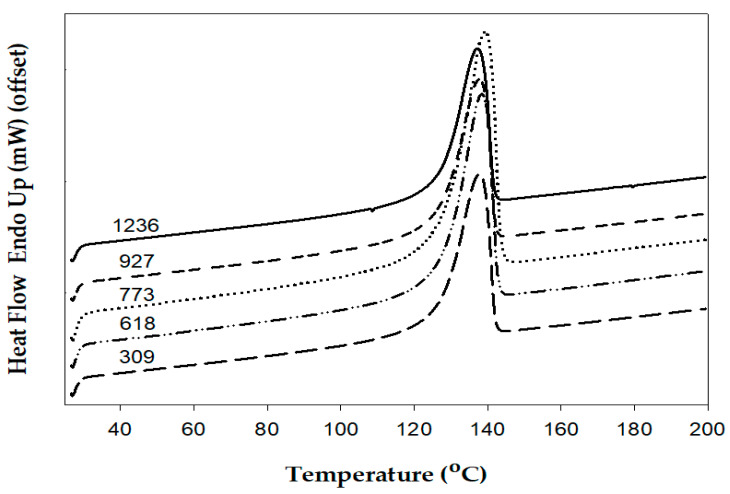
DSC second heating curves for polyethylene with various [Al]/[Ti] molar ratios.

To further investigate the influence of the cocatalyst/catalyst [Al]/[Ti] molar ratio on the thermal stability of homopolymerization, characterization of the obtained polymers was carried out through the use of TGA and DSC. Figure 5 indicates the TGA thermograms for five molar ratios of cocatalyst TEA to Ti [Al]/[Ti]: 309, 618, 773, 927, and 1236 mol. Table 2 shows the *T*_max_ of decomposition. The catalytic effects for 618, 927, and 1236 are higher than that for 309. The most active catalysis was observed for 773, with the earlier stages having a low decomposition process. Thus, a certain level of thermal stabilization is produced by the presence of catalysts, which dramatically accelerate the decomposition process at a certain temperature. The *T*_m_ values were determined by assigning the maximum endotherm peaks. Figure 6 and Table 3 represent the DSC thermogram with the melting points and ∆*H*_f_, whose increase depends on the [Al]/[Ti] molar ratios; it increases until the ratio reaches 773 moles, and then it begins to decrease. The limitations in the chain branches of the homopolymers were identifiable in the current study [12]. As noted by Smith et al. [31], slow polymerization processes relative to industrial-scale polymerization rates like in the case of slurry polymerization that is performed at room temperature, the growing chains’ local temperature is anticipated to be relatively less than the dissolution temperature of a given linear polyethylene within the medium of reaction which is at roughly 90 °C in heptane. Subsequently, considering the growing chain, a maximum of 36 CH_2_ units of the given macromolecule could be considered dissolved in the respective surrounding medium prior to commencement of crystallization. What is more, as noted by the authors, the outlined length was relatively below the expected distance of 140 CH_2_ units that are required in the overall formation of various entanglements within the polyethylene melts. As such, the resultant formed macromolecule is highly linear. The use of the required amount of cocatalysts is key to boosting the operation of the catalyst. The cocatalyst can reduce the alkylate metal transition, which initiates the first polymer chain through the elimination of impurities [32]. Active sites can be deactivated because of overreduction in the chain transfer reaction, which is key for catalytic activities [13].

### 3.2. Ethylene/1-Octene and Ethylene/1-Hexene Copolymerizations and Characterization of the Copolymers

The ethylene/1-octene and ethylene/1-hexene copolymerization behaviors were assessed after the behaviors of ethylene homopolymerization were compared. The appropriate dosage of the TEA cocatalyst in homopolymerization with the TiCl_4_ catalyst was [Al]/[Ti] = 773. A similar TEA cocatalyst dosage was utilized in both ethylene/1-hexane and ethylene/1-octene copolymerization. Nevertheless, the copolymers of ethylene with α-olefins had numerous benefits compared with other homopolymers. These benefits include larger process capacity, lower viscosity, and higher flexibility; hence, an investigation of the performance of the copolymerization of the aforementioned PVC/BuMgCl/Ti(OBu)_4_∙TiCl_4_ catalyst was important. Additional quantities of α-olefin were a determining factor in ethylene/α-olefin copolymerization because the addition of α-olefin can significantly impact its polymer microstructure and considerably affect the behavior of catalyst polymerization. Various quantities of 1-hexene and 1-octene were added in the consequent polymerization of the catalyst in the current study, and their influence on catalytic properties is shown in Table 4, which also shows noticeably enhanced activity with the PVC/BuMgCl/Ti(OBu)_4_∙TiCl_4_ catalyst. There was an initial increase in the catalytic activities before a gradual decrease as the concentration of the comonomer increased. For example, the amount of 1-hexene increased from 40 to 160 mmol, while the 1-octene quantity increased from 32 to 127 mmol, and optimum activity was attained at 64 and 60 mmol, respectively. These results show that the copolymerization of 1-hexene, 1-octene, and ethylene was strongly activated by the comonomer. Tuskaev et al. [23], Hongrui et al. [26], and Muñoz-Escalona et al. [33] have studied the notable preliminary enhancement in activity by α-olefin, which is known as the comonomer effect. These studies were grouped or categorized according to physical and chemical effects. Similar to the hydrogen effect, inserting ethylene into the Ti–H bond leads to the production of the Ti–C_2_H_5_ complex, which remains a dormant site via the β-agostic effect. Ti–C_2_H_5_ complex sites are not active for polymerization. On the other hand, inserting 1-octene or 1-hexene resulted in the formation of active sites of Ti–C_6_H_13_, Ti–C_8_H_17_, and Ti–C_6_H_13_. Additionally, the comonomer unit causes a donor effect, improving the activities of the catalyst. Conversely, the physical effect causes a decrease in the polymer’s crystallinity as a result of the insertion of the comonomer, leading to accelerated monomer diffusion. Nevertheless, a comonomer will have significant and diverse impacts on the reactions based on the types of catalyst and the conditions of the polymerization [34]. However, the mechanism for the comonomer effect in ethylene/α-olefin copolymerization over ZN catalysts remains unclear, warranting more studies that can offer a better description. In the present study, the process initiated by the Ti(OBu)_4_ catalyst illustrates the features found in olefin polymerization.

**Figure 7:**
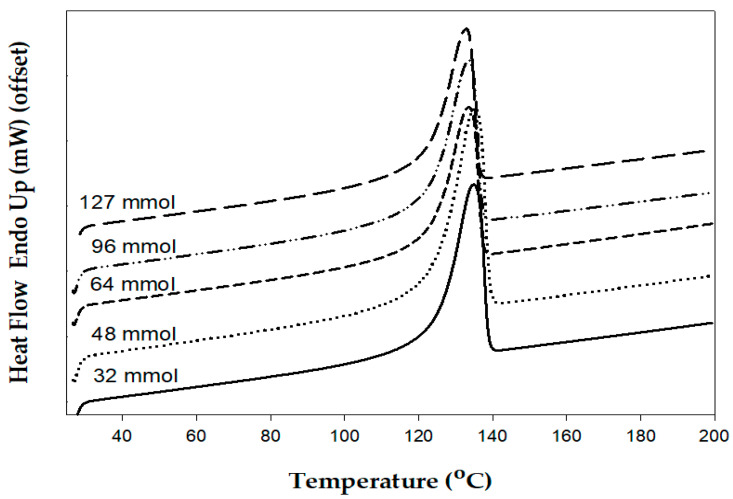
DSC second heating curve of the copolymer with various 1-octene concentrations.

**Figure 8:**
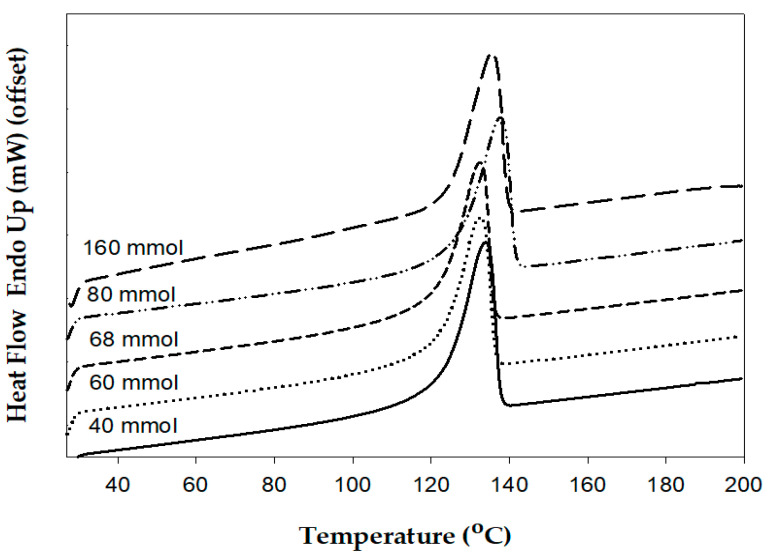
DSC second heating curve of the copolymer with various 1-hexene concentrations.

Table 4 shows a summary of the characterization of DSC. Figure 7 and Figure 8 show the respective second heating curves for ethylene/1-octene and ethylene/1-hexene. The findings reveal a reduction in the ∆*H*_f_ and the *T*_m_ of the copolymers because of the increased concentration of 1-hexene and 1-octene, as shown in Table 4; this occurred because of the insertion of a larger amount of comonomers in the mainstay of the polymer chain, which caused an increase in the concentration of comonomers. Based on the *T*_m_ values, the catalyst displayed lower sensitivity to 1-octene as the amount of 1-octene increased. The catalyst showed a greater sensitivity to 1-hexene as a result of the increased concentration of comonomer due to the introduction of the 1-hexene comonomer. The long chains caused an increase in T_m_ in addition to that caused by 80 and 160 mmol of 1-hexene because the configuration of chains became stiffer, leading to the appearance of additional extra small peaks in the melting line because of crystal gaps [35]. Furthermore, UHMWPE is believed to exhibit sharper incipient melting, which shows that the crystallinity difference between chains is smaller [35].

The present research utilized GPC to characterize the resultant copolymers. The findings reveal that the average *M*_w_ was reduced, except in the case of 68 mmol 1-hexene. There was an increase in the concentration of the 1-hexene to 160 mmol (up from 40 mmol), while the concentration of 1-octene increased to 127 mmol (up from 32 mmol). These findings are consistent with those of Yang et al. [36], who found that the strength of comonomer impacts tends to be greater in active centers, causing the production of a lower *M*_w_ polymer compared with those that produce higher *M*_w_ polymers; this then results in a substantial reduction of the average *M*_w_ of the entire polymer while its molecular weight distribution (MWD) is broadened. Furthermore, 1-hexene broadened the MWD more than PVC/BuMgCl/Ti(OBu)_4_∙TiCl_4_ catalysts did, and the polymerization of the 64 mmol of ethylene/1-octene demonstrated that the catalyst was highly balanced. The catalyst had the highest activity among the comonomers, while its PE products displayed the lowest MWD. Therefore, the lower sensitivity to 1-hexene for the catalyst is associated with the existing supports and the technique used for their preparation. In this case, the PVC-based polymeric support was the main support in the catalyst system. Thus, future studies should investigate the support used in catalyst systems.

**Figure 9:**
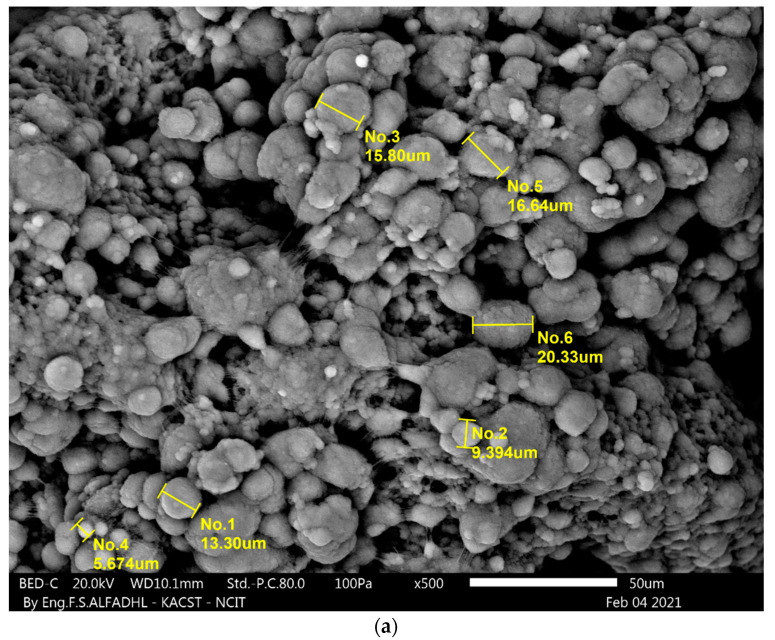

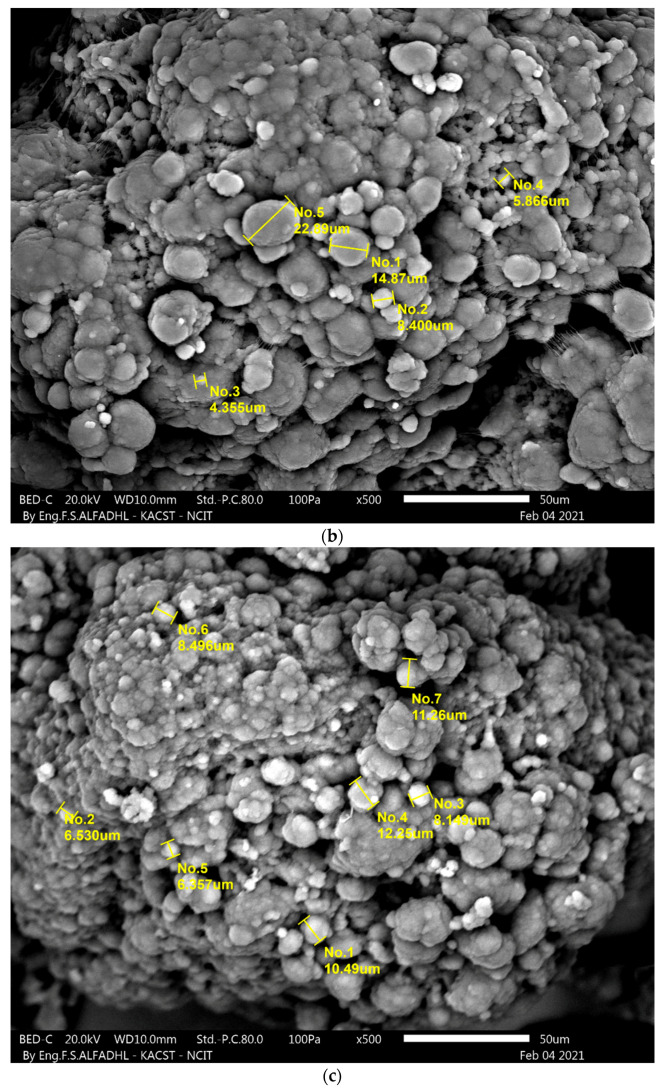
SEM images of the copolymer particles polymerized with catalyst using various 1-octene concentrations: (**a**) 32 mmol, (**b**) 64 mmol, and (**c**) 127 mmol.

SEM analysis of the comonomer concentrations was conducted to determine if there was a higher activity in ethylene/1-octene copolymerization. The results indicate that the catalyst particles had a satisfactory shape, showing increasing comonomer concentrations, as can be seen in Figure 9. (Details of the existence of all constituent atoms of the catalysts by analysis of EDS images are given in the Supplementary Materials, Figures S6–S8.) Moreover, a spherical copolymer was effectively disseminated or dispersed because it was replicated in the catalyst morphology. The copolymer particles grew gradually with increasing the 1-octene concentration, mainly because the catalyst particles in the preliminary polymerization phase break down into sub-particles and primary particles for easier separation. The primary catalyst particles initiate 1-octene/ethylene copolymerization, resulting in the formation of copolymer primary particles with sizes of 4–23 μm. Generally, these findings show that a PVC-based polymeric support has the capacity to allow for underground crystallization on the catalyst surface. The determining factor of the process of polymerization is the 1-octene concentration in n-hexane. These findings reveal that the preparation of the adduct solution containing a higher 1-octene concentration yields higher viscosity, preventing the dispersion of 1-octene, leading to its accumulation on external surfaces [37].

The contents of 1-octene together with 1-hexene relative to their incorporation of the copolymers as obtained from the catalyst were investigated using high-temperature ^13^C NMR. In this particular study, the copolymers chosen to be analyzed for incorporation were copolymers synthesized using 32, 64, and 127 mmol of 1-octene and 40 and 68 mmol of 1-hexene. The results of the analysis are outlined in Table 4 and Figure S9 illustrates the ^13^C NMR spectra within the Supplementary Materials. In this reaction, it can be asserted that too small quantities of 1-octene and 1-hexene may not affect the overall copolymerization behavior of given catalysts including copolymer’s microstructures while too large quantities of the comonomer may equally not be of benefit for the resultant activity. Consequently, the results of the study affirmed that having similar amounts of titanium species within a catalyst makes butoxide ligand beneficial for the overall insertion of 1-octene. The comonomer incorporation ability of respective hybrid ZN catalysts is affected prominently by titanium modification. As such, considering the initial stage, the molar ratio of 773 may be key in promoting the incorporation of 1-octene from 3.26% to 4.45% especially for Ti catalyst thus having 36.5% greater incorporation. There would be a pronounced difference in the 1-hexene incorporation thus there is the importance of controlling the comonomer content within the polymer.

## 4. Conclusions

Ti(IV) complexes that contain halogen atom alternatives such as Cl and are supported on PVC as a result of Grignard compound activation produce catalysts with better integration of 1-hexene and 1-octene. They also cause stronger activation of ethylene polymerization. An active catalyst was produced in the slurry homopolymerization of ethylene with the ZN catalyst, which was created using Ti(OBu)_4_. To obtain the highest activity, the optimum ratio of the cocatalyst TEA to Ti [Al]/[Ti] was 773:1, which resulted in higher activity. The higher the [Al]/[Ti] ratio, the smaller the molecular weight, suggesting that a higher [Al]/[Ti] ratio is associated with reduced *M*_w_. These findings illustrate that 1-octene integration attained the highest measured activity of 2.3 kg of PE/mol of cat∙h when fused with a 64 mmol copolymer. The increase in the concentration of 1-octene was found to be associated with a reduction in the catalytic activity in PVC/BuMgCl/Ti(OBu)_4_∙TiCl_4_. Both the ethylene/1-hexene copolymers and the catalyst demonstrated considerably lower activities. Therefore, the embodied catalyst has a polyethylene of higher *M*_w_, extensive MWD, and a negative comonomer impact with a consumption rate that is moderately or substantially unstable. Based on the SEM findings, the activated titanium sites played an important role in decreasing the crystallinity of the polymeric PVC. They also supported higher metal-active species on the inner surfaces of the support available to the comonomer in the slurry stage, which can be greatly increased with a rise in reaction activity. Moreover, the comonomer caused donor effects, leading to increased activity. From the standpoint of the physical impact, the generated copolymers caused a decrease in crystallinity, leading to the acceleration of monomer diffusion in the ethylene, which was assumed to be impassable in the crystallized polyethylene. The results and conclusions acquired in this investigation can be used as a guide to optimizing the synthesis of catalysts. Further, the experimental results of this study confirm theoretical studies that indicate the performance of active sites of ZN active sites having butoxide as a ligand of the titanium.

## Figures and Tables

**Table 1:** Influence of [Al]/[Ti] molar ratios on catalyst activity in ethylene polymerization.

[Al]/[Ti] Ratio	Catalyst Activity (g PE/g Cat. h)
309	700
618	800
773	1100
927	1050
1236	550

**Table 2:** Temperature for the maximum rate of decomposition obtained from the TGA thermograms of polyethylene with various [Al]/[Ti] molar ratios.

[Al]/[Ti] Ratio (mol/mol)	*T*_max_ (°C)
309	536.81
618	540.86
773	553.71
927	543.29
1236	544.95

**Table 3:** Melting temperature (*T*_m_) and enthalpy of fusion (∆*H*_f_) obtained from the DSC of polyethylene with various [Al]/[Ti] molar ratios.

[Al]/[Ti] ratio	*T*_m_ (°C)	∆*H*_f_ (J/g)
309	137.83	172.9197
618	138.4	262.5728
773	139.32	316.2398
927	137.72	179.0029
1236	137.25	203.4728

**Table 4:** Copolymer activities of different comonomers and polymer properties.

Sample	Amount of Comonomer (mmol)	Activity (g PE/mmol Cat. h)	*T*_m_^a^ (°C)	∆*H*_f_^a^ (J/g)	*M*w ^b^ (×10^5^ g/mol)	PDI ^b^	Comonomer Incorporation [mol%] ^c^
1-octene	32	1750	135	164.1	1.97	5.8	3.26
	48	2200	135	190.9	-	-	-
	64	2300	133	144.9	1.77	4.9	4.45
	96	2250	133	150.5	-	-	-
	127	2050	132	157.9	1.86	5.2	4.06
1-hexene	40	1500	134	151.3	2.47	7.3	1.34
	60	2000	133	144.2	-	-	-
	68	1900	132	143.2	3.26	4.9	4.87
	80	1650	138	161.3	-	-	-
	160	1450	136	147.5	2.31	6.8	-

Other polymerization conditions: cocatalyst [Al]/[Ti] molar ratio of 773:1. ^a^ *T*_m_ and ∆*H*_f_ calculated by DSC thermograms; ^b^ *M*_w_ and PDI estimated by GPC in TCB; ^c^ omonomer incorporated into copolymers estimated by high temperature ^13^C NMR and calculated according to the literature methods [24]. “-” Not determined.

## Data Availability

The data presented in this study are available on request from the corresponding author.

## Supplementary Materials

The following are available online at https://www.mdpi.com/article/10.3390/polym13132109/s1. Figure S1: SEM-EDS images of the ethylene polymerization carried out using [Al]/[Ti] molar ratio at 309. Figure S2: SEM-EDS images of the ethylene polymerization carried out using [Al]/[Ti] molar ratio at 618 mmol. Figure S3: SEM-EDS images of the ethylene polymerization carried out using [Al]/[Ti] molar ratio at 773 mmol. Figure S4: SEM-EDS images of the ethylene polymerization carried out using [Al]/[Ti] molar ratio at 927 mmol. Figure S5: SEM-EDS images of the ethylene polymerization carried out using [Al]/[Ti] molar ratio at 1236 mmol. Figure S6: SEM-Eds images of the ethylene/1-octene copolymerization carried out using 1-octene concentration at 32 mmol. Figure S7: SEM-Eds images of the ethylene/1-octene copolymerization carried out using 1-octene concentration at 64 mmol. Figure S8: SEM-Eds images of the ethylene/1-octene copolymerization carried out using 1-octene concentration at 127 mmol. Figure S9: High Temperature 13C NMR spectra of the copolymers: (a) 32 mmol of 1-octene, (b) 64 mmol of 1-octene, (c) 127 mmol of 1-octene, (d) 40 mmol of 1-hexene, and (e) 68 mmol of 1-hexene.
