# Supplementary material for: The Effect of Titanium Tetra-Butoxide Catalyst on the Olefin Polymerization
Source: Polymers (Basel). 2021 Jun 26;13(13):2109. doi: 10.3390/polym13132109 (PMC8271721; doi:10.3390/polym13132109)
Supplement: Supplementary file 1 [file polymers-13-02109-s001.zip › polymers-1282322-supplementary.pdf]

The Effect of Titanium Tetra-Butoxide Catalyst on the Olefin Polymerization

Mohammed S. Alsuhybani and Eid M. Alosime *

King Abdulaziz City for Science and Technology (KACST), P.O. Box 6086, Riyadh 11442, Saudi Arabia; sohybani@kacst.edu.sa (M.S.A.); alosimi@kacst.edu.sa (E.M.A.)

* Correspondence: alosimi@kacst.edu.sa

SEM – EDS analysis has been performed to study the catalyst particles and investigating the existence of element in ethylene homopolymerization and ethylene copolymerization.

Figures S1–S5 show the results of SEM-EDS analysis which have performed for ethylene polymerization tested at five different molar ratios of cocatalyst TEA to Ti [Al]/[Ti]: 309, 618, 773, 927, and 1236 mmol respectively.

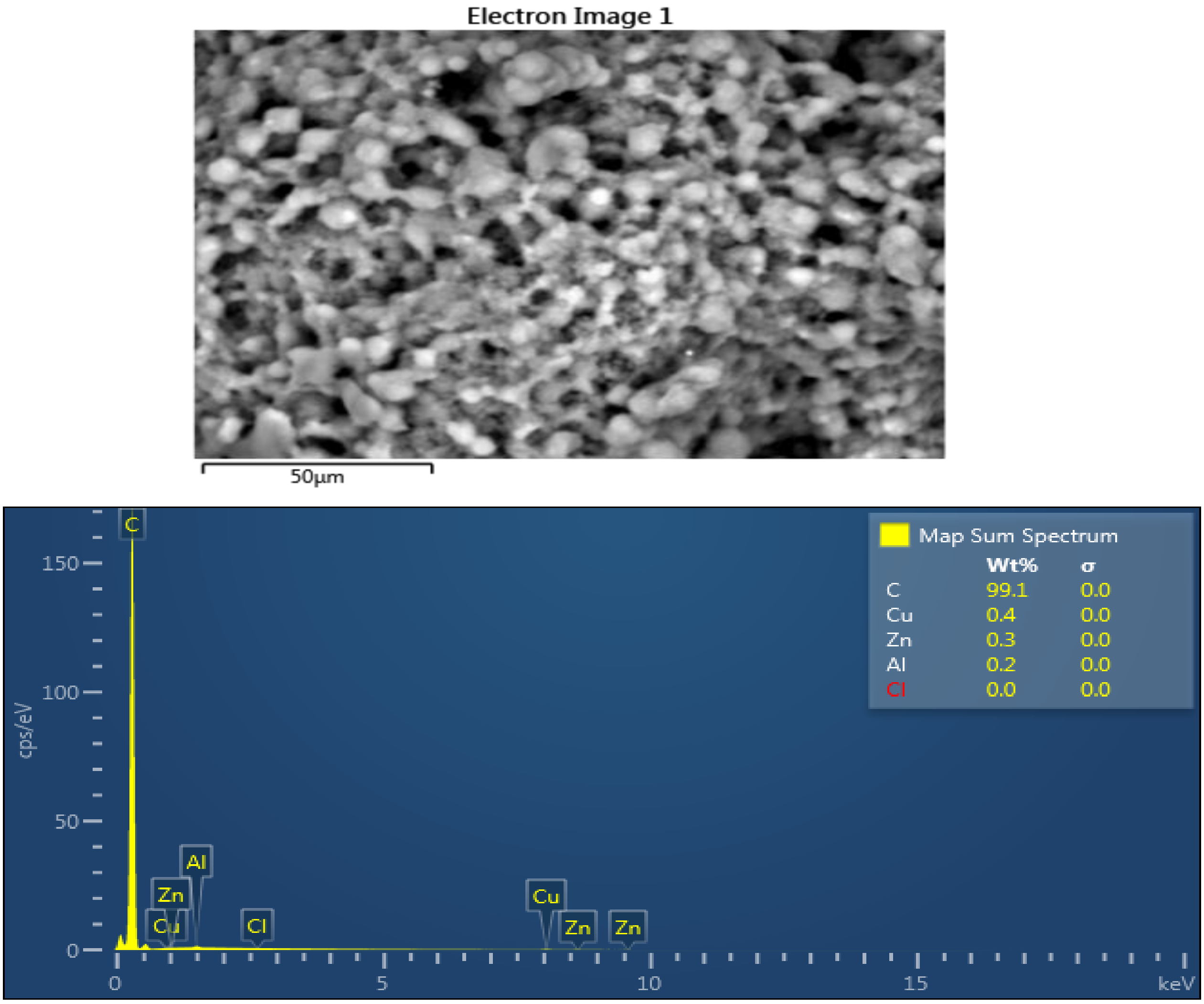

Element	Wt%	Wt% Sigma
C	99.12	0.02
Al	0.15	0.00
Cl	0.02	0.00
Cu	0.41	0.01
Zn	0.31	0.01
Total:	100.00	

Figure S1. SEM-EDS images of the ethylene polymerization carried out using [Al]/[Ti] molar ratio at 309.

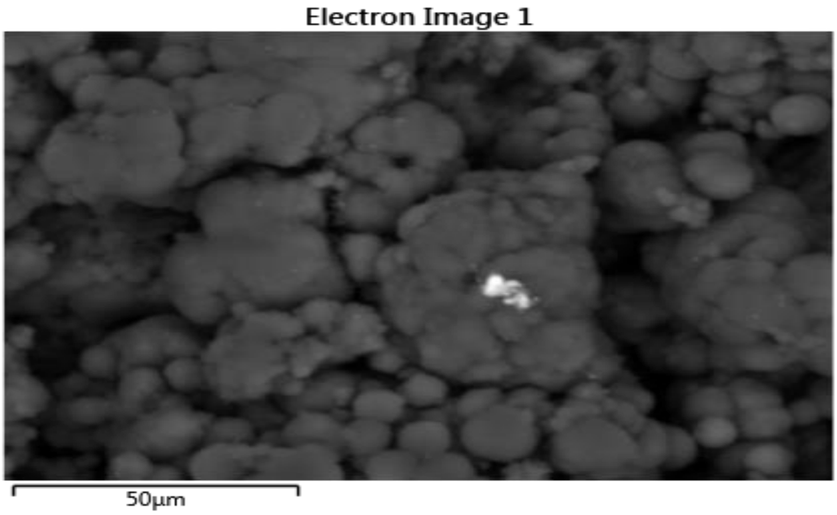

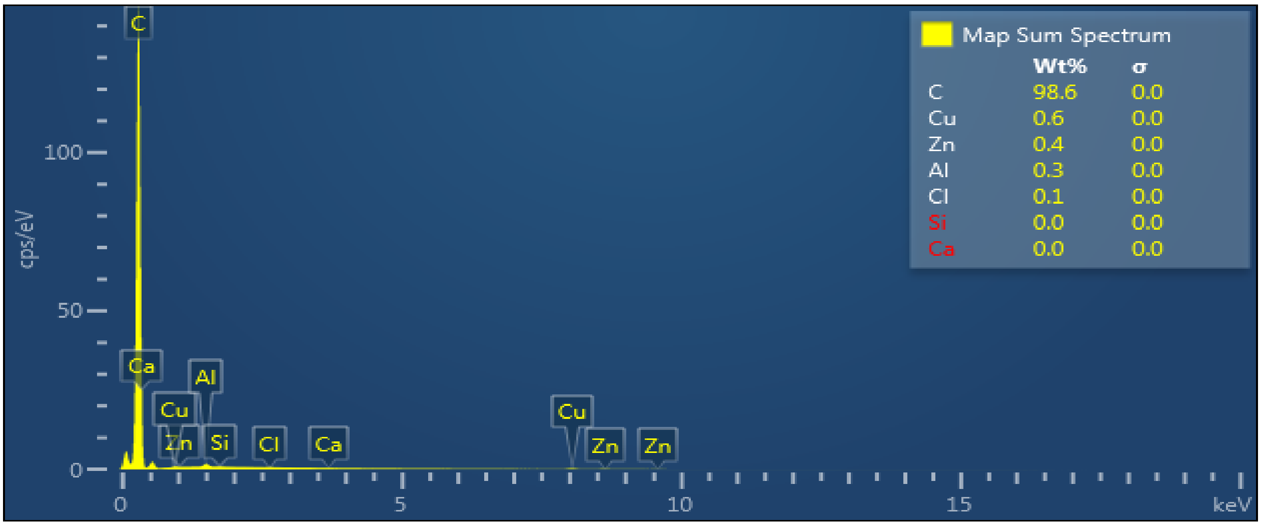

Element	Wt%	Wt% Sigma
C	98.62	0.02
Al	0.28	0.00
Si	0.03	0.00
Cl	0.05	0.00
Ca	0.02	0.00
Cu	0.57	0.01
Zn	0.43	0.02
Total:	100.00	

Figure S2. SEM-EDS images of the ethylene polymerization carried out using [Al]/[Ti] molar ratio at 618 mmol.

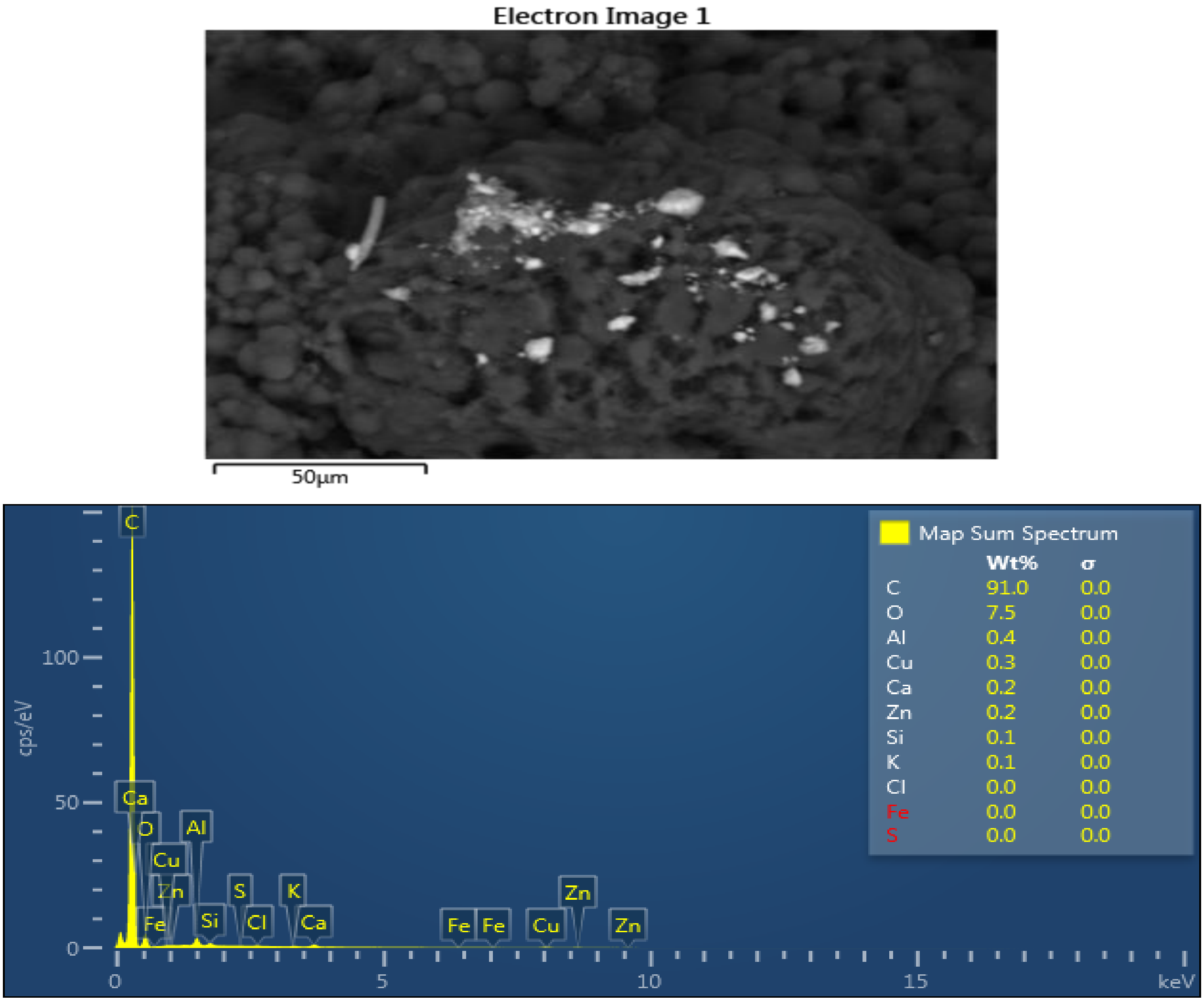

Element	Wt%	Wt% Sigma
C	91.00	0.03
O	7.53	0.03
Al	0.44	0.00
Si	0.12	0.00
S	0.01	0.00
Cl	0.05	0.00
K	0.06	0.00
Ca	0.23	0.00
Fe	0.04	0.00
Cu	0.32	0.01
Zn	0.20	0.01
Total:	100.00	

Figure S3. SEM-EDS images of the ethylene polymerization carried out using [Al]/[Ti] molar ratio at 773 mmol.

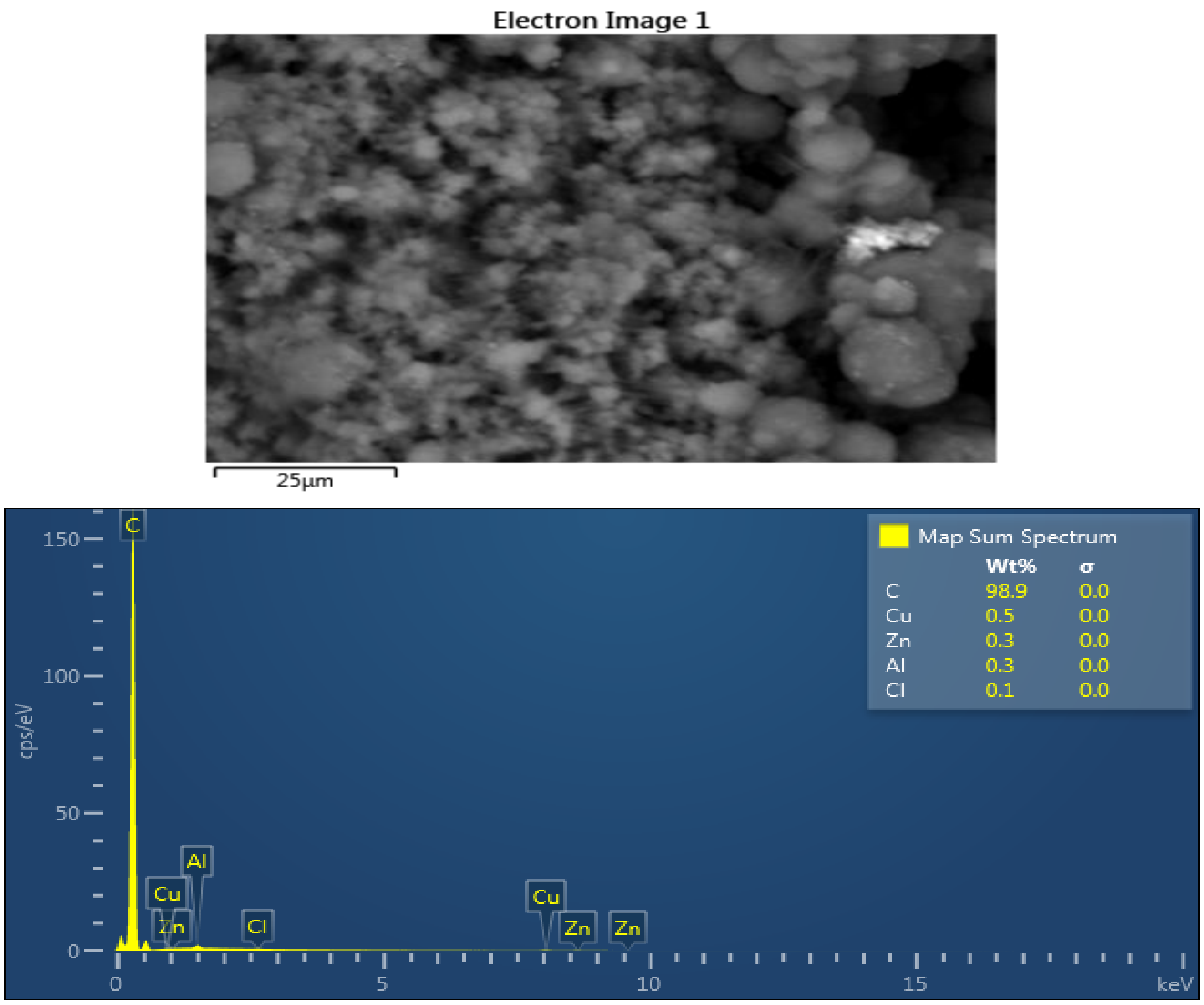

Element	Wt%	Wt% Sigma
C	98.85	0.03
Al	0.28	0.01
Cl	0.06	0.01
Cu	0.47	0.02
Zn	0.33	0.02
Total:	100.00	

Figure S4. SEM-EDS images of the ethylene polymerization carried out using [Al]/[Ti] molar ratio at 927 mmol.

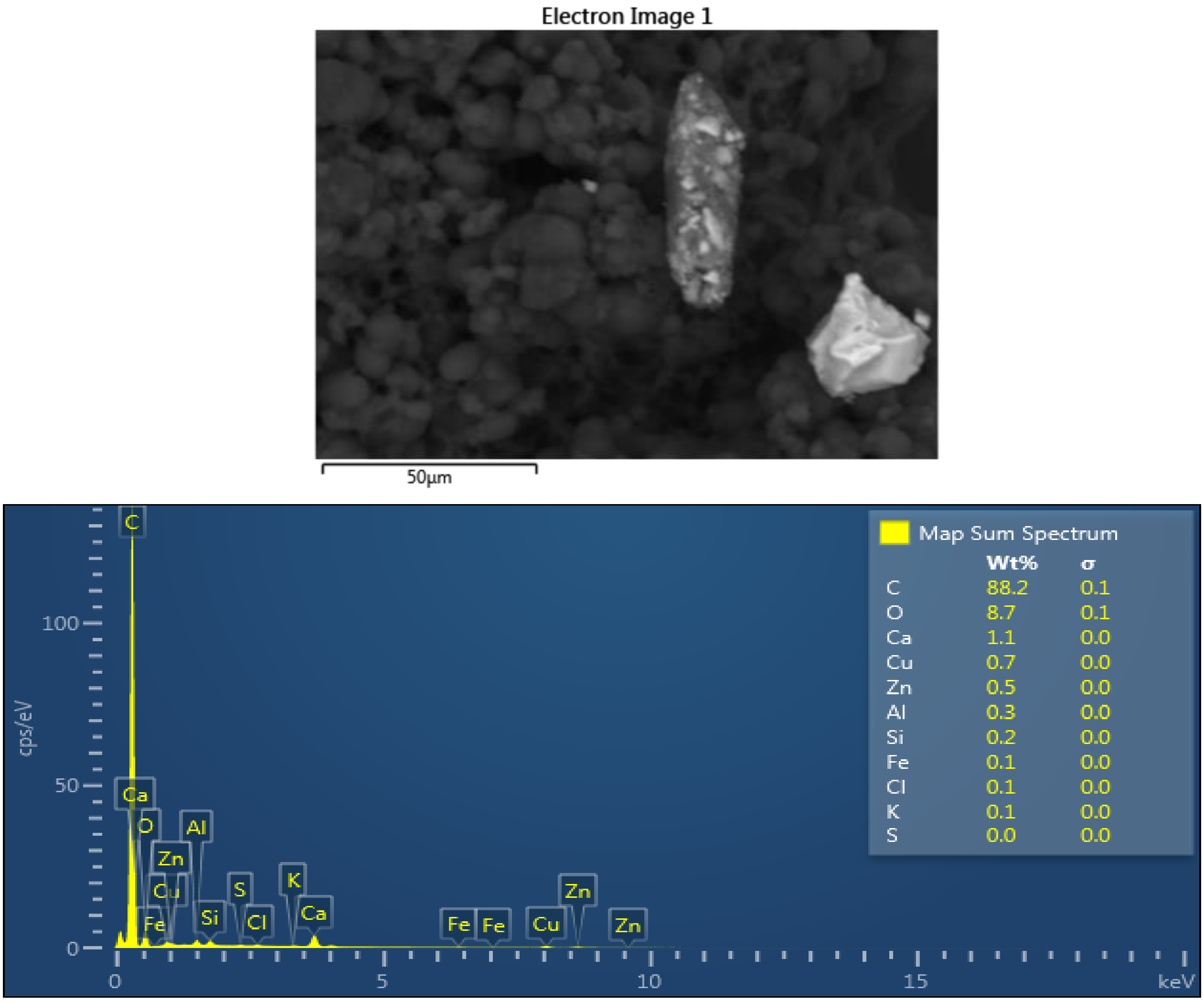

Element	Wt%	Wt% Sigma
C	88.16	0.06
O	8.70	0.05
Al	0.26	0.00
Si	0.23	0.00
S	0.05	0.00
Cl	0.08	0.00
K	0.07	0.00
Ca	1.12	0.01
Fe	0.08	0.01
Cu	0.74	0.01
Zn	0.51	0.01
Total:	100.00	

Figure S5. SEM-EDS images of the ethylene polymerization carried out using [Al]/[Ti] molar ratio at 1236 mmol.

Figures S6–S8 show the results of SEM-EDS analysis, which have performed for the ethylene/1-octene copolymerization with various 1-octene concentration 32 mmol, 64 mmol and 127 mmol respectively.

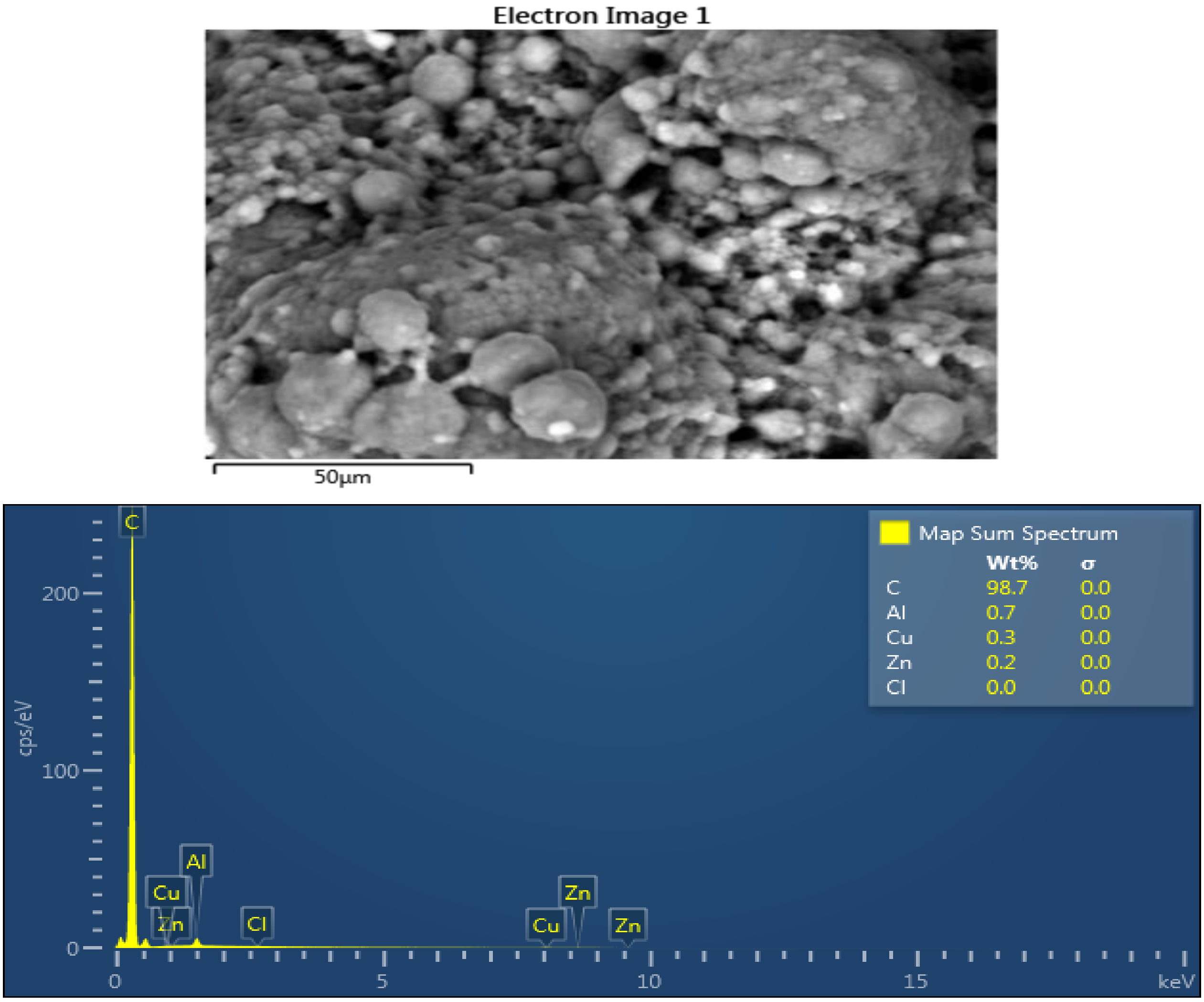

Element	Wt%	Wt% Sigma
C	98.69	0.02
Al	0.71	0.01
Cl	0.05	0.00
Cu	0.33	0.01
Zn	0.23	0.01
Total:	100.00	

Figure S6. SEM-Eds images of the ethylene/1-octene copolymerization carried out using 1-octene concentration at 32 mmol.

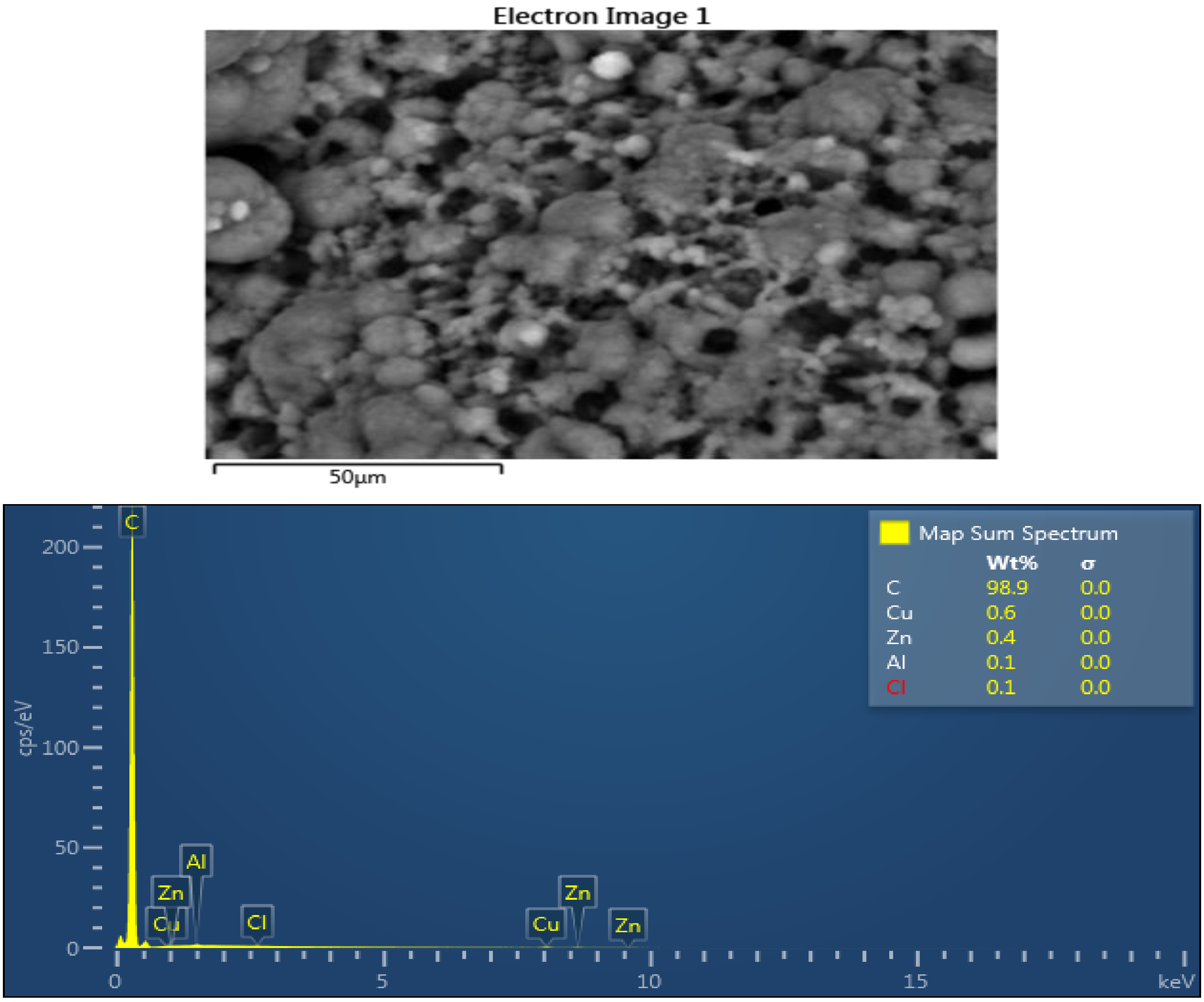

Element	Wt%	Wt% Sigma
C	98.85	0.04
Al	0.11	0.01
Cl	0.06	0.01
Cu	0.59	0.02
Zn	0.38	0.03
Total:	100.00	

Figure S7. SEM-Eds images of the ethylene/1-octene copolymerization carried out using 1-octene concentration at 64 mmol.

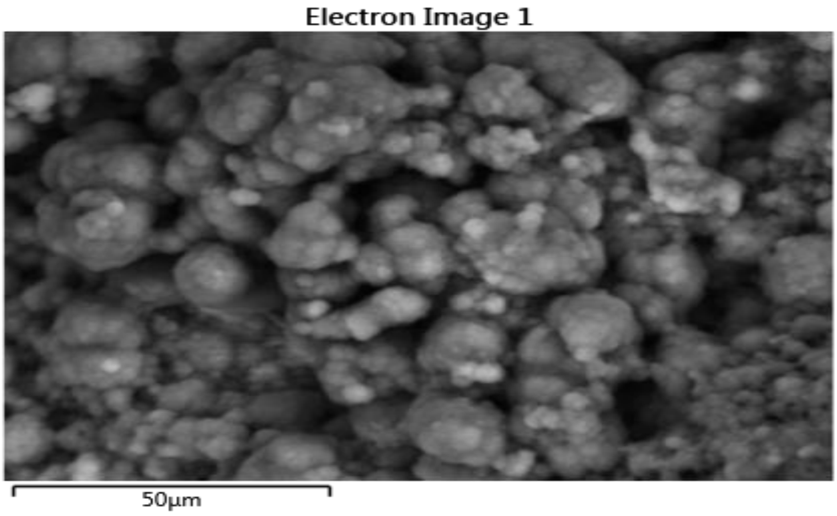

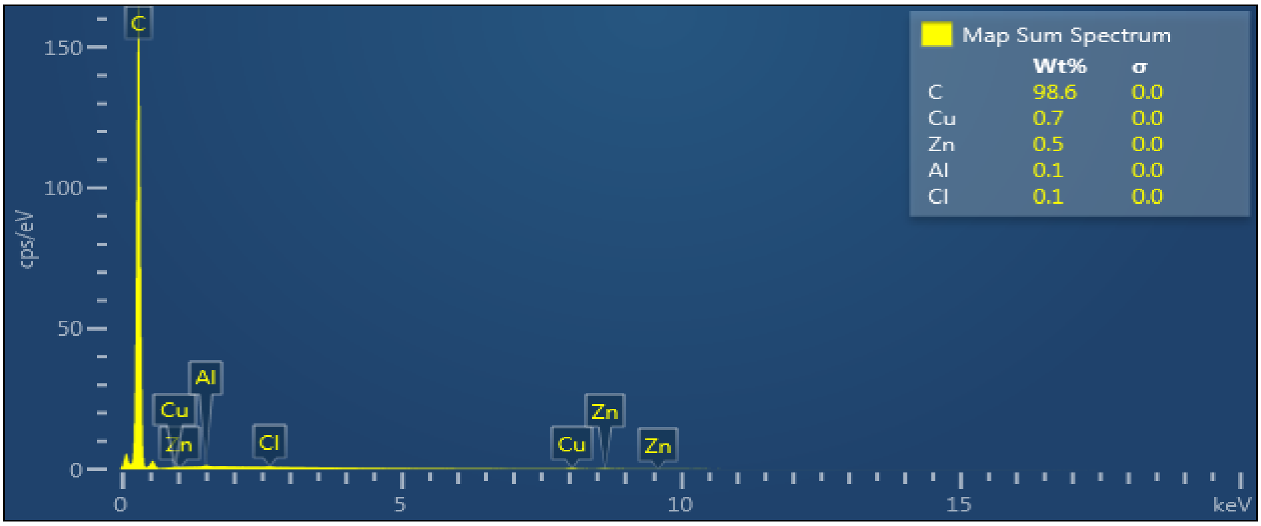

Element	Wt%	Wt% Sigma
C	98.55	0.03
Al	0.11	0.01
Cl	0.06	0.01
Cu	0.74	0.02
Zn	0.53	0.02
Total:	100.00	

Figure S8. SEM-Eds images of the ethylene/1-octene copolymerization carried out using 1-octene concentration at 127 mmol.

High temperature ^{13}C NMR has been performed to analyze the copolymers obtained from the prepared catalyst.

a)

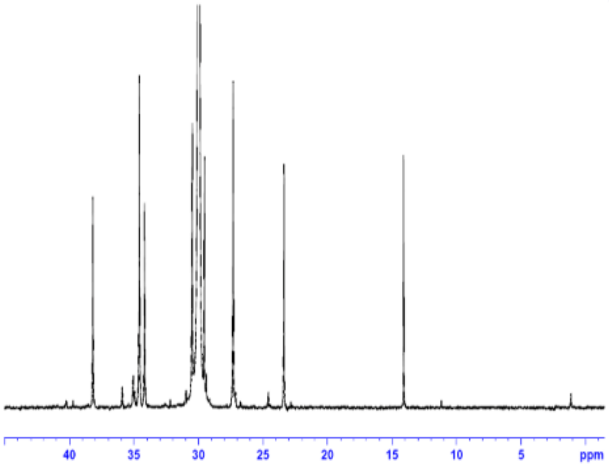

b)

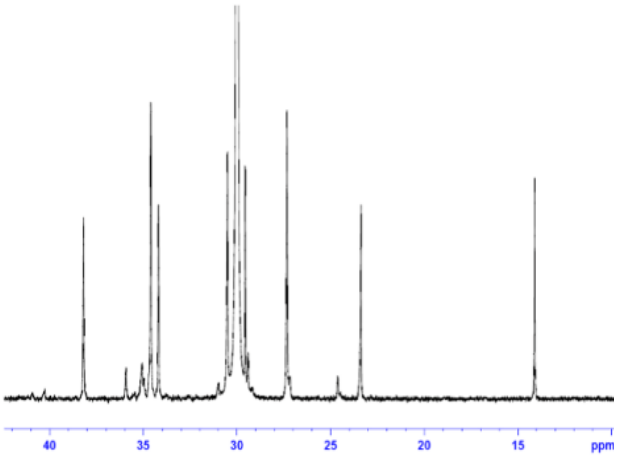

c)

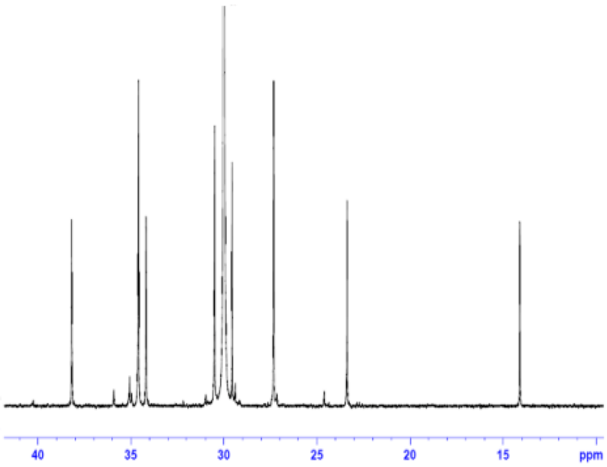

d)

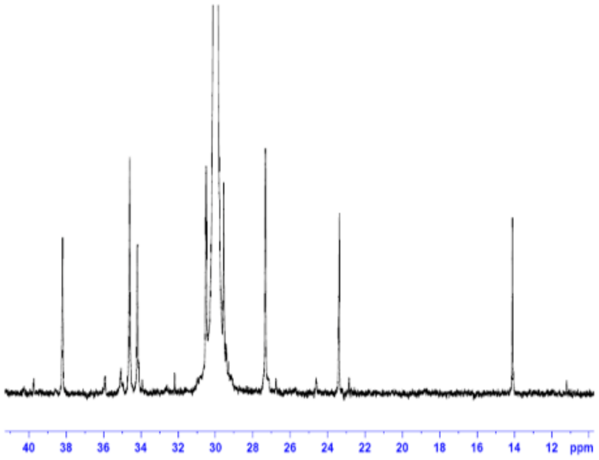

e)

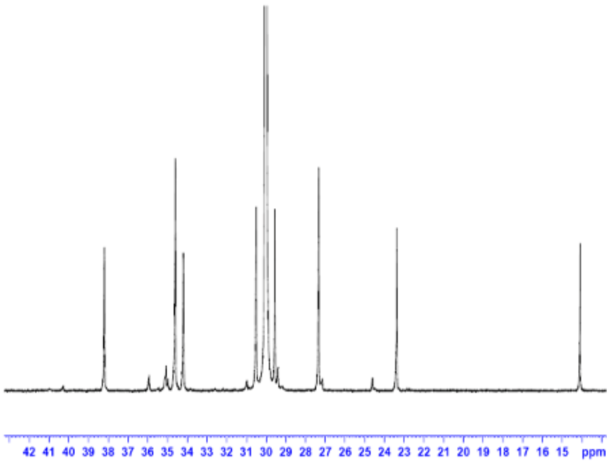

Figure S9. High Temperature 13C NMR spectra of the copolymers: a) 32 mmol of 1-octene, b) 64 mmol of 1-octene, c) 127 mmol of 1-octene, d) 40 mmol of 1-hexene, and e) 68 mmol of 1-hexene.
